# Effect of Hurdle Approaches Using Conventional and Moderate Thermal Processing Technologies for Microbial Inactivation in Fruit and Vegetable Products

**DOI:** 10.3390/foods11121811

**Published:** 2022-06-20

**Authors:** Aswathi Soni, Gale Brightwell

**Affiliations:** 1 AgResearch Ltd., Hopkirk Research Institute, Cnr University Ave and Library Road, Massey University, Palmerston North 4442, New Zealand; gale.brightwell@agresearch.co.nz; 2 New Zealand Food Safety Science and Research Centre, Massey University Manawatu (Turitea), Tennent Drive, Palmerston North 4474, New Zealand

**Keywords:** fruits, vegetables, D values, thermal, PEF, PATS, MATS

## Abstract

Thermal processing of packaged fruit and vegetable products is targeted at eliminating microbial contaminants (related to spoilage or pathogenicity) and extending shelf life using microbial inactivation or/and by reducing enzymatic activity in the food. The conventional process of thermal processing involves sterilization (canning and retorting) and pasteurization. The parameters used to design the thermal processing regime depend on the time (minutes) required to eliminate a known population of bacteria in a given food matrix under specified conditions. However, due to the effect of thermal exposure on the sensitive nutrients such as vitamins or bioactive compounds present in fruits and vegetables, alternative technologies and their combinations are required to minimize nutrient loss. The novel moderate thermal regimes aim to eliminate bacterial contaminants while retaining nutritional quality. This review focuses on the “thermal” processing regimes for fruit and vegetable products, including conventional sterilization and pasteurization as well as mild to moderate thermal techniques such as pressure-assisted thermal sterilization (PATS), microwave-assisted thermal sterilization (MATS) and pulsed electric field (PEF) in combination with thermal treatment as a hurdle approach or a combined regime.

## 1. Introduction

Thermal processing of food can be explained as any post-harvest process that uses heat to eliminate microbial contaminants (related to spoilage or pathogenicity) and extend shelf life using either microbial inactivation or/and by reducing enzymatic or toxin activity in the food [1]. However, consumer preference for minimally processed or “fresh like” food products have attracted significant research and development on mild to moderate thermal processing techniques. However, a hurdle approach that includes both thermal and non-thermal processing techniques has indicated the potential to increase food safety by microbial inactivation while reducing loss in nutritional and sensory attributes [2]. Fruits and vegetables are considered processed if they are cut/packaged in any form that is ready to eat by consumers. However, as soon as the fruit is cut or peeled, the possibility of microbial contamination from the surface or during handling increases [3]. These challenges have led to the use of processing techniques to minimize microbial contaminations that otherwise lead to spoilage and in some cases food poisoning. This review focuses on the use of thermal and hurdle approaches using various techniques to enhance food safety and also outlines the effects on sensory and nutritional quality. The technologies included in this review are pulsed electric field (PEF), pressure-assisted thermal processing (PATP) and microwave-assisted thermal sterilization (MATS).

## 2. Microbial Contaminants of Fruits and Vegetables

Fruits and vegetables remain an essential component of the human diet. Most countries across the globe face a regular, consistent and increasing demand for the production of fresh produce. While the annual production could efficiently cater for the population requirements, >20% of the total production has been reported to be lost due to spoilage [4]. Microbial contaminants of fruits and vegetables can lead to either food spoilage or food poisoning, therefore raising economic as well as health concerns. Post-harvest handling, packaging techniques and storage conditions as well as moisture content can all affect contamination and the survival of viruses on fresh fruits and vegetables. Once the fruits and green leafy vegetables are harvested and sorted to remove any damaged products, they are usually stored under conditions that prevent water loss and reduce any microbial proliferation [5]. It is crucial to maintain a constant, acceptable, optimal temperature, which is specific for each species of plants being stored, throughout harvest, storage and transport. Transportations are either via road/truck or via air through planes, which have their respective benefits and limitations [6]. For example, while transport via truck enables storage at temperatures and humidity within the limits of acceptability, the time taken from the harvest site to the destination could be long, especially for fruits with a short shelf life (<7 days) such as strawberries [6]. On the other hand, this time could be significantly reduced if transported via air, but it is rather challenging to maintain the optimum conditions of temperatures and humidity. Right from harvest to cutting, packaging and storage, there are possible routes for microbial contamination. Some of the common foodborne pathogens and spoilage-related microbial contaminants are listed in Table 1.

Therefore, while disinfection and prevention-based solutions against bacterial, viral and fungal contamination are useful for fresh produce with a limited shelf life, the storage of products with a fruit and vegetable base requires processing that involves mild–moderate thermal effect or some other inactivation mechanisms that can render the product free of any contaminants.

## 3. Conventional Thermal Processing Regimes of Fruits and Vegetables

Conventional thermal processing of fruits and vegetables can be divided into two major classes: pasteurization and sterilization. The key difference is based on the temperature and time of processing, according to which sterilization aims to remove all the bacterial contaminants including spores, whereas, in the case of pasteurization, spores might not be inactivated. While sterilization is the preferred regime for a long-term shelf life of food products, especially without the need to be stored under refrigerated conditions, pasteurization has the limitation of a limited shelf life. These applications enable the manufacturers of vegetable and fruit products to decide on the processing regimes that are fit for their purpose.

### 3.1. Sterilization of Fruits and Vegetables

Conventional thermal sterilization is explained by the United States Food and Drug Administration (FDA, Silver Spring, MD, USA) as any process using heat either alone or in combination with technologies that can lead to the inactivation of microorganisms including mesophiles and thermophiles to ensure that spoilage and food poisoning is eradicated [19,20]. The conventional method for evaluating the efficiency of thermal processing is dependent on the thermal value/lethality value or sterilization value F_0_ (F-value), which is then defined as the time (minutes) required to eliminate a known population of the resistant bacterial population in a given food under specified conditions [21,22]. It is also usually calculated as 12 D, which is the time needed for a 12 log reduction of thermally-resistant mesophiles, most commonly, *Clostridium botulinum* spores. *Clostridium sporogenes* have been used as the biological indicator for evaluating the microbiological efficiency of sterilization processes [23] due to their high thermal resistance and absence of any toxic genes, unlike *C. botulinum*. Thermal resistance is measured using decimal reduction time or the D value, which can be defined as the time required at any specific temperature to achieve inactivation equivalent to 1 log CFU/mL [24,25]. D values for *C. sporogenes* at 121 °C have been reported to be 0.5 min in phosphate buffer (pH 7.0) [26]. The thermal resistance of bacterial spores can vary significantly based on many factors. For example, the environmental conditions pertaining to both the spore induction and spore destruction process, the water activity and moisture content of the food being treated, and the pH, salt content and methods being used for D value assessments [27,28,29,30]. Therefore, based on the D value of the biological indicator spores in a specific type of food product, their F_0_ values are estimated. The F_0_ values for vegetable and fruit products could therefore vary significantly (Table 2), and due to limited publications associated with the intellectual property and commercial ownership, there are not much data available for reference.

Although there are a wide range of food matrices to be considered, a general classification for retorting dependent on the level of thermal treatment (time and temperature) is based on pH. Food products are classified as low acid or moderate to high acidic food products. Most of the fruit purees and fruit concentrates belong to the acidic group, where the pH of these products is generally around 2.5–3.5 [37]. However, a few exceptions include peach, where the pH is moderate (3.8–3.9) [38]. Any product above the pH 3.8 is considered low acid, where the risk of contamination and growth of *Clostridium* spp. is not controlled [39]. For these types of products, thermal exposure is at the coldest spot to ensure the reduction in 12 logs (12 D) of *C. botulinum*, for which an F_0_ of >3 min is recommended for an extended shelf life and food safety. While these treatments have ensured food safety, they have been reported to have a significant effect on the bioactive components as well as the sensory attributes [40,41].

### 3.2. Pasteurization of Fruits and Vegetables

Pasteurization was originally invented by French scientist Louis Pasteur, who invented the process of heating liquids at a temperature of about 55 °C for a short-defined time to eliminate bacterial contaminants [42]. With time, pasteurization became a common process in the dairy industry, and milk pasteurization can be either slow or fast. A slow process uses temperature–time combinations of 63 to 65 °C for over 30 min or 75 °C for 8 to 10 min. On the other hand, fast/rapid pasteurization uses a time–temperature combination of 85 to 90 °C or for up to 15 s [43]. Vegetables would in general be considered low acid foods and, therefore, need an efficient treatment to inactivate pathogens such as *L. monocytogenes*, which are pathogens of concern. *L. monocytogenes* has been reported to be present in either raw and minimally processed vegetables on multiple occasions, and the route of contamination is not completely known but is presumed to be soil, faeces or water [44,45,46,47]. The consumption of raw vegetables or fruits contaminated with *L. monocytogenes* results in listeriosis. The ability of *L. monocytogenes* to survive and grow in low/refrigerated temperatures (below 8 °C), increases the risk of foodborne listeriosis. Common symptoms include diarrhoea, fever, headache and myalgia (muscle pain); however, it has been reported to have a high mortality rate in pregnant women, infants and immunocompromised individuals with symptoms such as myalgia (muscle pain), septicaemia and meningitis [48,49,50]. This indicates the importance of the adoption of a zero-tolerance policy for *L. monocytogenes* by the FDA in the USA and the European Regulation on Microbiological Criteria for Foodstuffs, who have implemented a policy around the complete absence of *L. monocytogenes* for any food that is recommended for infants or special medical purposes [51].

In addition, spoilage-related bacteria are a concern to the shelf life of vegetables. Therefore, whether in the form of juices or purees, the process of treatment varies according to the type of vegetable/fruit, moisture and most importantly pH. There are not many studies that have reported on the effect of the pasteurization of vegetable purees or fruit juices due to commercial sensitivity. Most of the research published has included pasteurization as a comparative standard method to see the efficiency of non-thermal technologies. For example, a study by Kathiravan, et al. [52] reported the effect of various combinations of time and temperature for pasteurization on bioactive components as well as the inactivation of native microflora in beetroot juice. The results indicated that pasteurization at 96 °C for a total heating time of 720 s resulted in the maximum retention of bioactive compounds such as betacyanin and betaxanthin while inactivating the native microflora [52]. It has been reported that juices (for example, cantaloupe juice and watermelon juice) can result in cross-protection to heat due to acid stress, thereby increasing the D values of the foodborne pathogens such as *Salmonella*, *E. coli* O157:H7 and *L. monocytogenes* [53]. Another study reported similar heat resistance in *E. coli* O157:H7 E0139 in acid-adapted apple cider and orange juice, thereby resulting in an up to two times increase in D_52_ °C values [54]. Therefore, it can be concluded that the time and temperature combination of pasteurization could be dependent on the fruit and vegetable matrix, including characteristics such as moisture content and pH, and the intrinsic resistance of the bacterial species being targeted. While bacterial spore formers are known to have higher D values as compared to the non-spore forming vegetative bacterial strains, there is a significant difference in the D values among different strains of same bacterial species in various food products. Table 3 indicates a few examples of *L. monocytogenes* and *E. coli* to support and reflect the diversity in thermal resistance in vegetative cells in various food matrices.

Thermal processing efficiency can be significantly challenged due to the natural biodiversity and therefore their thermal resistance [54], and this challenge further increases in ready-to-eat food products, where the bacterial thermal resistance could be different with each product, including the significant difference among those in meat versus dairy versus vegetable purees. Therefore, the combination of more than one technology (including thermal) might provide a better assurance of inactivation.

## 4. Alternative Approaches Involving Moderate Thermal Treatment and Hurdle Approaches for Fruits and Vegetables

The complex challenge of ensuring food safety, along with an attempt to preserve the maximum fresh-like attributes of fruits and vegetable products, has led food manufacturing companies to invest in research associated with mild to moderate thermal interventions that could be combined with non-thermal techniques to deliver similar lethality to that of pasteurization or, in some cases, sterilization. While there is no single alternative to thermal technologies/sterilization, using a combination of more than one technique, such as irradiation + heat, pressure + heat, electroporation + heat and microwave processing + heat, has recently gained significant attention due to their promising potential.

### 4.1. Pulsed Electric Field (PEF) Treatment and Thermal Processing

**Figure 1:**
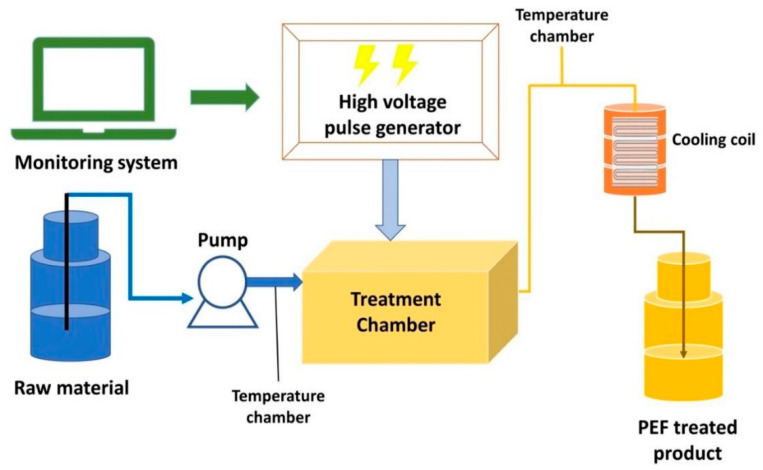
Diagram of a possible continuous PEF device used to treat food samples (adapted from Taha, et al. [64] (reprinted from Pulsed Electric Field: Fundamentals and Effects on the Structural and Techno-Functional Properties of Dairy and Plant Proteins. Taha, Ahmed, Casanova, Federico Šimonis, Povilas Stankevič, Voitech Gomaa, Mohamed A. E. Stirkė, Arūnas; Foods 2022; Vol. 11; Issue 11 Page 1556 under a Creative Commons license)).

PEF treatment of food involves the dispatch of short pulses of short and high voltage to achieve electric field strengths of 15–35 kV/cm at specific energies (50–700 kJ/kg) through the food to induce the formation of pores in the outer membrane of microbial cells [61]. The application of field strengths between the electrodes leads to the formation of transmembrane potential differences over the cellular membrane, thereby leading to pore formation, which could be either reversible or irreversible [62,63]. When this potential difference exceeds a critical value, pore formation occurs in the membrane of the cells. A schematic diagram of a PEF device in food processing as reported by Taha, et al. [64] is represented in Figure 1.

Although PEF is considered a non-thermal technology for the extraction of bioactive food components from fruit and vegetable products, microbial inactivation has only been reported to be successful with the use of moderate temperatures (<50 °C) [65,66]. Table 4 indicates a few examples where PEF and heat have been reported against bacterial pathogens in fruits and vegetables. For bacterial inactivation, the increase in membrane permeability, cytoplasm conductivity and instability of the electrochemical state in the cell membranes are needed to render the damage irreversible. For this level of injury in the bacterial cells, the potential difference needs to be high, as indicated in Table 4. The cell wall has been reported to be the main target for disrupting the integrity and morphology of *Bacillus pumilus* cells when PEF (1000 pulses of 5 μs from 2 to 7.5 kV/cm) was applied [67]. The changes also indicated an increase in osmotic pressure inside the cell and damage to the cell wall and, specifically, to the peptidoglycan structures/chains. Moreover, spores were found to be more resistant to these changes due to their rigid external protective cortex [67]. A study by Soni et al. reported the upregulation of a gene called chitooligosaccharide deacetylase, which is associated with peptidoglycan degradation, when *B. cereus* spores were treated using PEF at 80 °C [68]. However, there is a lack of evidence in the literature that reveals the structural damages associated with PEF and heat on bacterial pathogens in food, although several studies have reported inactivation (Table 4).

PEF treatment has many benefits over using conventional pasteurization or sterilization. For example, the use of moderate heating minimizes the loss of organoleptic properties, as well as prevents denaturation of the heat-sensitive vitamins and bioactive compounds in fruits and vegetables. However, the use of PEF for the inactivation of bacterial spores has not been successful unless the overall treatment (pre or post or during) of the system reaches more than 80 °C [68,72]. In this study by Soni et al., an overall temperature increase of ~17 °C was observed, and a separate control was included to see the effect of this temperature increase on thermal resistance and the inactivation of the spores. It was observed that a temperature increase did not lead to any inactivation or loss of thermal resistance, and the observed results were an output of the combination of PEF and pretreatment at 80 °C [68]. The resistance of bacterial spores to PEF (stand-alone treatment) can be attributed to the outer structure of the spores consisting of the cortex and coat, which prevents electroporation, unlike vegetative cells.

There are a few limitations that have prevented PEF being extensively used in the fruit and vegetable industries. The first limitation is around the high cost and energy requirements for the generation of the high-voltage pulses that are required to deliver sufficient power to process products in large quantities as well as in a continuous application [61]. Based on the cost analysis by Toepfl et al. [73], the economic cost of the PEF treatments to improve the phenolic extraction from grape mass could be around 0.01 and 0.2 EUR/t to deliver the energy inputs of up to 6.76 kJ/kg [61]. For microbial inactivation, that is equivalent, when compared to pasteurization, to the treatment either having to employ high electric field strengths between 25 and 35 kV/cm for a longer treatment time or to use a pretreatment with mild to moderate heat, which can further increase the energy inputs and, hence, the overall cost. However, PEF has been successful in improving the extraction of phenolic compounds in fruit mass (juice or wine) and in controlling spoilage microorganisms as long as the initial bacterial load is not high [61,74,75]. However, further research on using hurdles to reduce the cost of PEF processing to achieve 5–6 Log CFU/mL of non-spore-forming bacterial populations is required.

### 4.2. Pressure-Assisted Thermal Processing (PATP)

**Figure 2:**
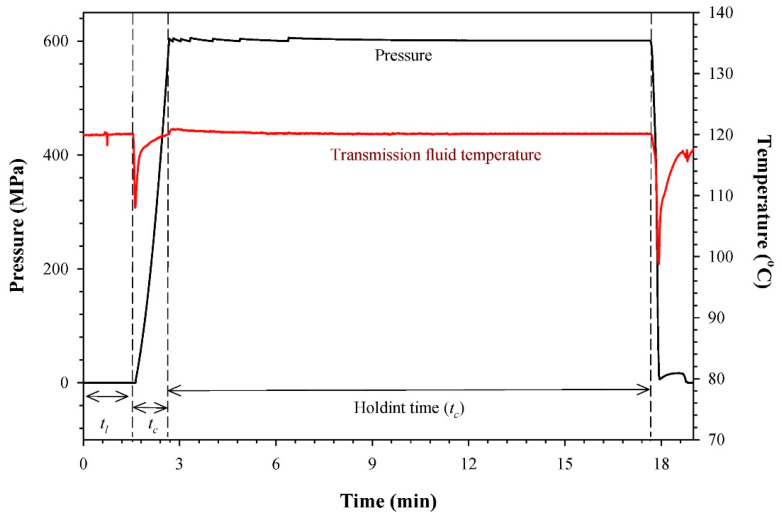
A graph indicating typical pressure, temperature and time history during pressure-assisted thermal processing of conjugated linoleic acid (CLA)-enriched milk treated at 600 MPa/120 °C (tl, loading time; tc, compression time; th, holding time [78]) (reprinted from Combined Effect of Pressure-Assisted Thermal Processing and Antioxidants on the Retention of Conjugated Linoleic Acid in Milk. Martinez-Monteagudo, Sergio I., Saldaña, Marleny D.A.; Foods 2015; Vol. 4; Issue 2 Page 65–79 under a Creative Commons license).

PATP is a food processing method that combines the effect of pressure (600 to 900 MPa) and heat (90 to 121 °C) to inactivate bacterial pathogens in food with a reduced effect on heat-sensitive nutrients [76,77]. In comparison to the conventional heating (retorting or pasteurization) process, PATP is known to reduce the processing time due to the mechanism of adiabatic compression due to applied pressure (Figure 2).

Pressure-assisted thermal sterilization (PATS) is a type of PATP, which is also accepted by the Food and Drug Administration (FDA, Silver Spring, MD, USA) in the U.S as a thermal sterilization regime for shelf-stable low acid food products ((pH > 4.6) [79]. PATS uses a temperature over 100 °C and high pressure above 600 MPa to inactivate bacterial spore formers in food and generate a shelf-stable product with maximum retention of the organoleptic properties and nutrients [80]. These terms have been interchangeably used in the literature to indicate the combined use of thermal processing and high-pressure processing. The mechanism of action during bacterial inactivation by PATS is a synergistic combination of high temperature and high pressure to generate an adiabatic compression heat, which in turn affects the cellular architecture of the bacterial cell [77]. This further leads to functional damages in the cell such as an increase in cell membrane permeability, alerted structure and confirmations of the organelles modifications in the biochemical reactions, and, ultimately, cell death [81]. The alteration of the cell membrane structure through damage to proteins and the phospholipids bilayer, and therefore loss of the integral composition, has been known to be the major cause of cell death [82]. This process is accelerated by the high temperature, which alters the structural conformation of the proteins and lipids involved in the cellular structures or functions [83].

Although several studies in the literature have highlighted the nutritional retention by using PATP, Table 5 includes specific studies that reported the use of PATP for bacterial inactivation in fruits and vegetables.

A study by Thai Nguyen, Rastogi and Balasubramaniam [84] compared the effect of thermal processing and PATS on the reduction/inactivation of the background flora by using a non-selective media for enumeration and incubating the plates aerobically at 37 °C. These results were supported by the previous work [90] on a non-food matrix where up to a 6 log reduction in *B. amyloliquefaciens* using PATS 700 MPa–121 °C < 1 min was achieved. However, as specific bacterial strains were not used for challenge testing, and as there could be a significant difference in the thermal resistance of bacterial strains (especially spores), there could be variation in the results obtained. Another study specifically tested the background flora in pumpkin puree for the presence of *Clostridium perfringens* and *Bacillus* spores alongside non-spore-forming strains such as *Escherichia coli.* The results indicated that up to 2 log CFU/mL of *Bacillus* spores were inactivated and all the non-spore-forming strains were below the detection limit after treatment at 900 MPa and 80 °C for 1 min [86]. The studies on the inactivation of bacterial spores by PATP (Table 4) indicated that the significantly resistant forms of spores such as *B. amyloliquefaciens* and *G. stearothermophilus* could also be reduced by 3.1 and 4.8 log CFU/mL. The extension of nutrient retention has been individually reported where possible (Table 5) indicating a reduced loss in bioactive (ascorbic acid, carotenoids and phenolic) compounds.

PATP has already been commercialized and is used for a processing treatment to deliver lethality equivalent to the pasteurization of fruit and vegetable soups, juices and purees. However, there exists a challenge of achieving the compression heating that is able to hold the required pressure to complete the treatment in an insulated setting so that the heat and pressure can simultaneously be used for achieving maximum microbial inactivation in a minimum time [91,92]. In addition, the cost of achieving a high pressurization rate, using a vessel material with low heat-transfer properties such as polyoxymethylene (POM) or polyether ether ketone (PEEK), and installing an internal intensifier system to prevent heat losses is another of the challenges faced by many food industries [93]. As per a cost analysis (capital cost, labour, maintenance and depreciation along with equipment cost), the total cost was reported to be ~USD 0.0455/lb to install PATS in an existing line and produce a product [92].

### 4.3. Microwave-Assisted Thermal Sterilization (MATS)

**Figure 3:**
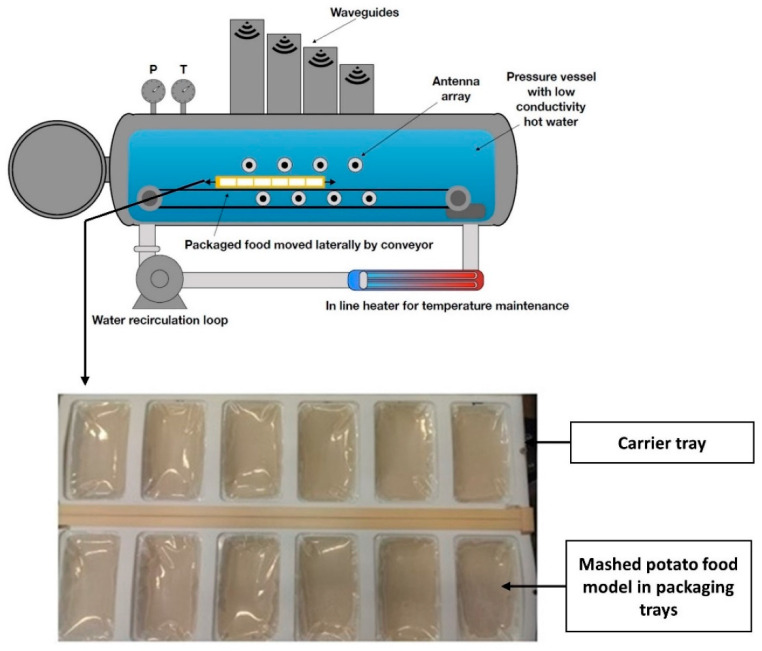
Schematic representation of batch processing model for microwave sterilization, also known as coaxially-induced microwave pasteurization and sterilization (CiMPAS), where the packaging trays are arranged in carrier tray while kept immersed in hot/warm water inside the pressure vessel [25]. (Reprinted from Development of Bacterial Spore Pouches as a Tool to Evaluate the Sterilization Efficiency—A Case Study with Microwave Sterilization Using *Clostridium sporogenes* and *Geobacillus stearothermophilus*. Martinez-Monteagudo, Sergio I., Saldaña, Marleny D.A.; Foods 2020; Vol. 9; Issue 10, Page 1342 under a Creative Commons license.)

MATS technology employs a combination of both thermal (convection) and microwave energy (conduction) to sterilize food in polymeric packages to ensure microbial inactivation that is considered equivalent to thermal sterilization; however, with reduced loss of sensitive nutrients unlike thermal sterilization [19,94]. MATS has also been approved by the Food and Drug Administration (FDA, Silver Spring, MD, USA) for the sterilization of homogeneous and non-homogeneous foods [94]. In this process, the food (in polymeric packages) is kept submerged in hot water at 121 °C and treated using microwaves (915 MHz) under pressurized hot water to achieve the desired F_0_ to eliminate bacterial contaminants [94]. This technology is relatively new and is mostly used for ready-to-eat food products that have vegetable and fruit portions. A schematic representation of this technology is shown in Figure 3.

The benefit of MATS over thermal sterilization is its reduced processing time, which can thereby reduce nutrient loss. Microbial inactivation for commercial sterilization aims at an F_0_ of 3 (Table 1) or more [95]. A few studies that have reported success in achieving a more than 6 log reduction in bacterial spore formers in fruit or vegetable matrices are listed in Table 6.

The exact mechanism of action of microwave processing (2450 MHz) has been debatable due to multiple concepts. For example, it has been postulated as the thermal effect of microwave exposure [98,99], but the non-thermal effects of the radiation on bacterial cells have also been reported [100,101]. On the other hand, the process of microwave-assisted thermal sterilization (915 MHz at 121 °C) has been postulated to be a completely thermal process, and the mechanism of action, as with all thermal inactivation, is via structural damage of the cellular components of bacterial cells and spores [19,94,102]. However, there is an evident lack of studies reporting any structural changes in bacterial cells due to microwave sterilization.

Microwave sterilization offers several benefits over conventional methods; however, there are a few challenges yet to overcome. Since water is not the heating medium in this process of microwave sterilization, the temperature remains stable at a preset point, and therefore any over-processing or increase in the final temperature of the processed product can be reduced. However, the process of microwave-based heating is largely dependent on the dielectric property of food, especially the dielectric constant and dielectric loss factor, which determines the behaviour and interaction of food with electromagnetic fields [103,104]. Dielectric properties are further dependent on the moisture, salt and fat content of the food product [105] and further on the temperature and frequency used in the treatment [94]. The challenge of non-uniform temperature distribution in conventional household microwave ovens has been overcome by the microwave sterilization regime to a great extent [106]. A study by Soni et al. identified cold spots in a mashed potato model using Maillard browning products. For verification of microbial inactivation at these spots, *C. sporogenes* spores were inoculated on these predetermined cold spots using spore pouches followed by recovery and enumeration post-treatment at 121 °C (6 passes, 12 kW) at 915 MHz. The results indicated that a more than 6 log reduction in spores was obtained at each cold spot, ensuring food safety [25]. Microwave processing at 915 MHz also offers the potential of processing at lower temperatures for products that would require thermal treatment equivalent to pasteurization, therefore offering the potential for fruit processing. The specific packaging material or polymeric trays/pouches are a cost of the food processing companies, and recycling options are under research but are not yet defined [19,107]. In-line installation of microwave sterilization units in the existing processing line could have a significant cost; however, a cost analysis has not been reported.

## 5. Conclusions and Future Perspective

Thermal sterilization and pasteurization of fruit and vegetable products offer the safest options for microbial food safety. However, they also lead to significant losses in nutrients such as Vitamin C and bioactive compounds such as carotene, which makes the food shelf-stable but also less nutritious. For the purpose of achieving the target of sterilization as well as nutrient retention, novel technologies have now been combined with thermal treatment to achieve maximum lethal value in the minimum time of exposure. The hurdle approach or a combination of more than one technique to ensure microbial inactivation could contribute toward a medium to longer shelf life and nutrient retention. Many such treatments were discussed in this review and some are as of yet underinvestigated, and promising technologies have been highlighted such as MATS. MATS and PATP show significant potential but need more investigation at temperatures below 100 °C to ensure at least a 6 log reduction in spore formers. Although PEF has emerged as an effective tool for the extraction of bioactive compounds from plant cells, it has not proven to be successful in the inactivation of bacterial spores in food unless combined with temperatures above 100 °C. Moreover, pumpable fruit products such as purees and juices have proven to be more successful at being processed using PEF as compared to solid products.

While these novel technologies including PEF and PATP have long been investigated against conventional processing, MATS remains in its early stage of research. Their industrial implementation is challenging due to more than one reasoN. For example, the design of the instrumentation might significantly differ from one laboratory/manufacturer to another, therefore making the direct comparison of results very difficult. As these novel technologies are currently being majorly investigated as a research tool in pilot-scale laboratories, their production costs, as well as their feasibility for integration in line with existing techniques, are either higher than conventional thermal technologies or are unclear. Furthermore, if these technologies replace the conventional methods, the overall impact on the environment, the economy, energy consumption and food wastage might be linked to acceptance by consumers.

Therefore, further research on using the novel processing techniques at an industrial scale followed by investigations on the impact of their usage in the social, economical and financial areas will increase their sustainable applications in the food industries.

## Figures and Tables

**Table 1:** Common contaminants of fruits and vegetables.

Microbial Contaminants (Bacterial/Viral/Fungal)	Relevance	Food Safety/Shelf Life-Based Concerns	Reference
*Salmonella* spp. (serovar Typhimurium, Montevideo, Javiana, Anatum, Enteritidis, Infantis, Stanley, Newport)	Foodborne pathogen resulting in self-limiting gastroenteritis in humans. Multidrug resistance is well known	Has been reported as a common cause of food poisoning in many countries; fresh produce can be contaminated anytime from harvest to packaging	[7,8]
*E. coli* O157:H7	Foodborne pathogen resulting in haemorrhagic colitis, bloody diarrhoea hemolytic uremic syndrome and death	Cross-contamination from meat during the preparation of ready-to-eat (RTE) products has been reported. Multiplication and growth of *E. coli* on fresh produce are reported at 12–25 °C	[9,10]
*Campylobacter jejuni*	A foodborne pathogen that causes gastroenteritis	Outbreaks associated with fresh salads have been reported. Although cross-contamination has been reported as one of the causes, many sources of contamination remain unidentified	[11,12]
*Listeria monocytogenes*	Food poisoning resulting in mild gastroenteritis to severe blood and/or central nervous system infections with limited reports on abortion in pregnant women	*L. monocytogenes* is a contaminant of fresh produce and can also be prevalent in RTE and minimally processed meals	[13]
*Aeromonas* spp.	Food poisoning leading to gastroenteritis	Due to their ubiquitous nature, they contaminate the vegetables and fruits via fresh and salt water, either during harvest or post-harvest handling	[14]
*Pseudomonas* spp.	Opportunistic pathogens are known to be capable of producing pathogenicity factors (toxins, effector proteins, proteases, elastases and pigments) that might affect the immune system. Otherwise associated with spoilage	Mishandling during harvest or post-harvest leads to cross-contamination from Pseudomonas coming from the soil, fertilizers, manure or water used for irrigation	[15]
Hepatitis A virus	The causative agent of hepatitis A leads to mild to moderate symptoms and fatality in some cases. Additionally known to cause frequent endemics in developing countries	Fruits and vegetables can be cross-contaminated if irrigated with water/solutions that contain faecal remains.	[16]
Norovirus	Associated with foodborne outbreaks and usually referred to as stomach flu. It leads to diarrhoea, vomiting, nausea and stomach pain	Usually, cross-contamination during handling and packaging and also due to exposure to faecal cross-contaminants.	[17]
Mycotoxins: *Aspergillus* spp., *Penicillium* spp. and *Alternaria* spp.	Associated with food poisoning and spoilage and significant loss of the harvest products	Post-harvest contamination by *Aspergillus* spp., *Penicillium* spp. and *Alternaria* spp. causes toxin production as part of their secondary metabolites and in some cases leads to spoilage such as citrus brown spots by *Alternaria alternata*.	[18]

**Table 2:** Sterilization value or F_0_ for vegetables and fruits.

Composition of the Product	F_0_- Approximate Sterilization Value/Range (min.)	References
Asparagus	F_121_ = 3	[31]
Carrot puree	F_121_ = 4.9	[32]
Celery pure in a stew	F_121_ = 8	[33]
Green beans in brine	F_121_ = 6	[34]
Canned gudeg (jackfruit and spices) in coconut milk	F_121_ = 28	[35]
Onions in calcium brine	F_121_ = 6	[36]
Peach low acid canned food	F_93_ = 3	[37]

**Table 3:** D values of non-spore-forming bacteria.

Bacterial Species and Food Matrix	D_70_ Values in Specific Matrix (min)	Reference
*L. monocytogenes* in milk	3.0 ± 0.5	[55]
*L. monocytogenes* F4243 in meat	0.13	[56]
*L. monocytogenes* in duck muscle/meat	0.11 ± 0.01	[57]
*L. monocytogenes* in soya bean product	0.95	[58]
*E. coli* O157:H7 in soya bean product	3.94	[58]
*E. coli* O157:H7 in apple juice	0085	[59]
*E. coli* in ground beef	1.3	[60]

**Table 4:** Effect of PEF + moderate heat on bacterial inactivation in fruit/vegetable products.

PEF Parameters/Settings	Product	Bacterial Inactivation Potential	Effect on Bioactive Compounds	Reference
Electric field strength of 2 kV/cm, the pulse width of 1 μs with a frequency of 100 pulses per second at 31 °C for 6 min	Blueberries in salt solution	3 log reductions of *E. coli*, *Listeria innocua*, separately	10% and 23% increase in anthocyanins and total phenolics, respectively	[69]
Electric field strength of 25 kV/cm, 280 μs, 112 pulses and 767 Hz at a maximum temperature of 68 °C	Fresh mixed orange and carrot juice (80% orange and 20% carrot)	2.67 ± 0.61 reduction in total viable counts	75.6% reduction in pectin methylesterase activity, which otherwise leads to a reduction in the commercial value of the juice through loss of turbidity	[70]
Electric field strength of 25 kV/cm, 330 μs, 132 pulses and 904 Hz at a maximum temperature of 70 °C	Fresh mixed orange and carrot juice (80% orange and 20% carrot)	2.85 ± 0.30 reduction in total viable counts	81% reduction in pectin methyl esterase activity	[70]
Electric field strength of 34 kV/cm at specific energy of 650 kJ/L, frequency of 25 Hz for 150 s	Fruit Smoothie made up of pineapples, bananas, apples, oranges and coconut milk	6.9 log10 CFU/mL reduction in *Escherichia coli* K12	Not monitored	[71]
Electric field strength of 20 kV/cm, specific energy of 150 kJ/L, bipolar pulses of 25 μs	Orange Juice	5.6 log reduction in *E. coli* (ATCC 11775)	No significant loss in compounds (fresh flavour (e.g., dl-limonene, β-myrcene, α-pinene, and valencene)) attributing to the fresh-like sensory attributes	[66]

**Table 5:** Effect of PATP with heat on bacterial inactivation in fruit/vegetable products.

Product and PATP Parameters/Settings	Effect on Bioactive Compounds	Bacterial Inactivation Potential	Reference
Carrots (cylindrical pieces) treated at 500 to 700 MPa and the temperature range of 95 to 121 °C for up to 2 min	As compared to thermal treatment, PATS was ~70% more efficient at the retention of carotenes and therefore the colour.	The natural flora was inactivated beyond the detection limit of 10 CFU/unit.	[84]
Mango (*Mangifera indica* L.) pulp at 600 MPa at 52 °C for 10 min	Ascorbic acid, phenolics and antioxidant potential of mango pulp were not significantly affected and therefore were considered retained.	5 log inactivation (aerobic mesophiles, total coliforms, lactic acid bacteria)	[85]
Pumpkin puree at 900 MPa/80 °C	carotenoids and phenolic compounds in the puree were retained while polyphenol oxidase (PPO) enzyme activity was significantly reduced	4 log reduction in aerobic Colony Counts, 2 log reduction in total coliforms, and 2 log reduction in *Bacillus* spores	[86]
Green pee puree (pH 6.1) ohmically treated (50 V/cm) was treated at 600 MPa and 105 °C for 10 min	Not reported	2.5 and 3.9 log cfu/mL for *B. amyloliquefaciens* and *G. stearothermophilus* spores	[87,88]
Tomato juice (pH 4.1) ohmically treated (50 V/cm) at 600 MPa and 105 °C for 10 min	Not reported	3.1 and 4.8 log cfu/mL reduction in *B. amyloliquefaciens* and *G. stearothermophilus* spores, respectively, in tomato juice	[87]
Carrot puree (pH 5.0 ohmically (50 V/cm) treated at 600 MPa and 105 °C for 10 min	Not reported	2.80 and 4.11 for B. amyloliquefaciens and *G. stearothermophilus* spores	[87]
Mashed carrots treated at 800 MPa, 70 °C	Not reported	5.0 log/mL reduction in *B. amyloliquefaciens* spores	[87,89]

**Table 6:** Effect of microwave-assisted/induced sterilization with heat on bacterial inactivation in fruits/vegetable products.

Parameters/Settings	Product	Bacterial Inactivation Potential	Reference
Microwave-assisted thermal pasteurization at a frequency of 915 MHz, microwave power of 18.7 kW where the food package was moved at a speed of 116.8 cm/min under circulating water at 72 °C	Green beans	9.0-log CFU/g reduction in *L. innocua* ATCC 51742	[96]
Coaxially-induced microwave sterilization at 915 MHz, microwave power of 22 kW, where the food package was moved back and forth at a speed of 130 cm/min under circulating water at 121 °C for a total processing time of 68.3 min	Mashed potato	1–2 log CFU/g and >6 log CFU/g for *Geobacillus stearothermophilus* and *Clostridium sporogenes* spores, respectively	[25]
Continuous-flow microwave heating operating at 915 MHz, microwave power of 60 kW, preheated by pumping hot water at 130 °C and recirculating it for approximately 30 min (F_0_ = 5.13)	Sweet potato puree	4.85 × 10^6^ log CFU/mL reduction in *Bacillus subtilis* spores	[97]

## Data Availability

Not applicable.
